# Three-Dimensional Posture Analysis-Based Modifications After Manual Therapy: A Preliminary Study

**DOI:** 10.3390/jcm14020634

**Published:** 2025-01-19

**Authors:** Fabio Scoppa, Andrea Graffitti, Alessio Pirino, Jacopo Piermaria, Federica Tamburella, Marco Tramontano

**Affiliations:** 1 Chinesis I.F.O.P. Osteopathy School, 00152 Rome, Italy; fabioscoppa@chinesis.org (F.S.); graffittiandrea@gmail.com (A.G.); 2 Faculty of Medicine and Dental Surgery, University of Rome “Sapienza”, 00185 Rome, Italy; 3 Department of Biomedical Sciences, University of Sassari, 07100 Sassari, Italy; axelpir@uniss.it; 4 Santa Lucia Foundation IRCCS, 00143 Rome, Italy; jacopo.piermaria@gmail.com; 5 Department of Life Sciences, Health and Health Professions, University Link Campus of Rome, 00165 Rome, Italy; 6 Department of Biomedical and Neuromotor Sciences (DIBINEM), Alma Mater University of Bologna, 40138 Bologna, Italy; marco.tramontano@unibo.it; 7 Unit of Occupational Medicine, IRCCS Azienda Ospedaliero-Universitaria di Bologna, 40138 Bologna, Italy

**Keywords:** osteopathic manipulative treatment, gentle touch intervention, posture, cervical spine, lumbar spine

## Abstract

**Background/Objectives:** Manual therapies like Osteopathic Manipulative Treatment (OMT) and Gentle Touch Intervention (GTI) are widely employed for improving posture and spinal alignment, but their effects as measured using advanced technologies remain underexplored. This study aims to evaluate the short-term postural effects of these interventions using a non-invasive three-dimensional rasterstereography-based approach, focusing on the cervical arrow, lumbar arrow, kyphotic angle, and lordotic angle parameters. **Methods:** A three-armed randomized controlled trial was conducted with 165 healthy participants. The subjects were divided into control (CTRL), OMT, and GTI groups. Their postural parameters were assessed pre- and post-intervention using the Spine3D system by Sensor Medica (Guidonia Montecelio, Italy). The statistical analyses included paired *t*-tests and an ANOVA, with the significance set at *p* < 0.05. **Results:** Significant reductions in the cervical arrow were observed in both the OMT (*p* < 0.005) and GTI (*p* < 0.05) groups, while the kyphotic angle significantly improved only in the GTI group (*p* < 0.05). No significant changes were found in the lumbar arrow or the lordotic angle across the groups. The control group showed no postural variations, reinforcing the specificity of the interventions. **Conclusions:** Both OMT and GTI influence spinal posture, particularly in the cervical and thoracic regions. GTI, with its gentle approach, demonstrated unique effects on the thoracic curvature, suggesting neurophysiological mechanisms. These findings highlight the potential of manual therapies for posture modulation and suggest future research should explore their long-term benefits and applications in clinical populations.

## 1. Introduction

Among topography-based technologies, rasterstereography has garnered particular attention from the research community. Rasterstereography is a radiation-free, non-invasive imaging technique that provides a three-dimensional reconstruction of the dorsal surface. Its primary applications include postural assessment and spinal deformity screening. Although radiation-free systems are unable to directly assess the internal shape of the spine due to their reliance on evaluating the dorsal surface, they are considered a viable alternative to traditional radiographic imaging [1,2]. Rasterstereography typically provides an approximate 3D reconstruction of the spine, with its primary advantages being its cost-effectiveness and rapid execution, all without exposing the patient to ionizing radiation. As a result, rasterstereography has been widely adopted as a screening tool for the early detection of spinal deformities, particularly scoliosis [3,4]. In recent decades, various instruments have been developed for applying the principles of rasterstereography, with the first introduced by Drerup and Hierholzer in the 1980s [5].

Monaro and colleagues addressed the excellent intra-day and inter-day reliability in almost all of the parameters analyzed when using the Spine3D non-invasive three-dimensional optoelectronic detection system, suggesting it as a reliable tool capable of discriminating between different positions of the spine. They recommended it as an easy and fast way to analyze the surface shape of the spine for follow-up in clinical settings, as well as because the results in using the Spine3D methodology are similar to those found in studies with stereophotogrammetric systems [6], allowing us to consider this methodology able to measure such parameters. In line with these findings, we adopted the Spine3D system to test the postural changes induced by manual therapies, specifically Osteopathic Manipulative Treatment (OMT) and Gentle Touch Intervention (GTI).

Manual therapies, especially those targeting musculoskeletal structures, have been associated with both short-term and long-term modifications in posture and spinal alignment. While OMT is a well-established, hands-on technique that aims to diagnose and treat somatic dysfunctions through targeted manipulations [6,7,8,9], GTI involves subtle, non-invasive contact, designed to enhance proprioception and promote relaxation without applying a direct mechanical force. Both interventions are commonly employed in clinical settings to manage musculoskeletal pain, improve mobility, and enhance overall well-being [7,8,9]. However, their respective impacts on spinal posture, particularly when measured using advanced, non-invasive technologies like Spine3D, are less understood.

This study aims to evaluate the short-term postural effects of these interventions using a non-invasive three-dimensional rasterstereography-based approach. By assessing specific postural parameters (cervical arrow, lumbar arrow, kyphotic angle, and lordotic angle), we aimed to determine how these interventions influence the spinal curvature and postural alignment on the sagittal plane over a short-term period.

## 2. Materials and Methods

### 2.1. Participants

This study was conducted as a three-armed randomized controlled trial (RCT). The protocol was written according to the Declaration of Helsinki and approved by the Independent FSL Ethics Committee (Prot. Number CE/2023_029, approved on 9 May 2023). Written informed consent was obtained from all of the participants according to the FSL ethical procedures before their participation. The recruitment document explained that participation was voluntary, without incentives for the participants, and dependent on the inclusion and exclusion criteria. All interested participants received information about the project via telephone and were briefly interviewed by a clinician who was not involved in the intervention sessions to assess their eligibility according to the inclusion and exclusion criteria reported below. The inclusion criterion was as follows: an age between 18 and 45 years. Participants were recruited through the database of the Chinesis I.F.O.P. Osteopathy School of Rome. The exclusion criteria included the following: (i) Cognitive impairment, based on a Mini Mental State Examination (MMSE) [7] score ≤ 24 according to the norms for the Italian population [8]. (ii) Current or reported medical illnesses, e.g., diabetes (not stabilized), obstructive pulmonary disease, or asthma; hematologic and oncologic disorders; pernicious anemia; clinically significant and unstable active gastrointestinal, renal, hepatic, endocrine, or cardiovascular system diseases; or newly treated hypothyroidism. (iii) Current or reported orthopedic or neurological disorders. (iv) Not experiencing pain based on a Visual Analogue Scale score of less than 3 for at least 6 months before enrollment. (v) Pregnancy.

The participants were randomized into three groups using IBM SPSS Statistics V. 29 (Statistical Package for the Social Sciences—Chicago, IL, USA): the control (CTRL) group, the OMT group, and the GTI group. The randomization was performed through a block randomization model according to a computer-generated pseudo-randomized list. The participants were unaware of the study design and outcomes, as well as the group allocation. A researcher who was not involved in the intervention sessions collected the participants’ data and performed the randomization and was the only responsible for this process and securely stored the randomization list. A Case Report Form (available in Supplementary Materials) containing demographic information and designed to verify the absence of exclusion criteria was used to collect these data. Each participant underwent two testing sessions, spaced 48 h apart (S1: first session; S2: second session). The treatment sessions were performed by two healthcare professionals who had completed a training program in osteopathy aligned with the Italian Core Competencies in osteopathy [9] and with the European Standard on Osteopathic Healthcare Provision. Somatic dysfunctions were addressed according to the tissue alteration, asymmetry, range of motion, and tenderness (TART) parameters, which guided the osteopathic evaluation and intervention [9,10,11,12]. Somatic dysfunctions were detected in the whole body and then balanced one by one to define the primary order of treatment according to the TART parameters. For each participant, osteopaths used the outpatient osteopathic SOAP (Subjective, Objective, Assessment, Plan) note form. OMT techniques were focused on correcting the dysfunctions found during the initial physical examination and included articular and myofascial techniques, balanced ligamentous tension, visceral manipulations, and osteopathy in the cranial field [13]. GTI was performed by the same osteopaths who performed the OMT and was carried out in the same setting. GTI consisted of tactile stimulation at a precise force (0.2 N) and velocity using manual techniques [14]. Passive soft touch was applied to the lumbar, dorsal, and cervical spine; shoulders; hips; sternum; and chest without joint mobilization. The interventions were performed with the participants lying on the medical bed and the osteopaths standing next to the bed. Each intervention lasted 45 min.

The participants in the CTRL group were evaluated after a two-day interval, without receiving any treatment between S1 and S2. The individuals in the OMT group underwent OMT based on a global approach [15].

To determine a sufficient sample size power, an analysis was conducted using G*Power based on a previous paper [6]. Using an alpha equal to 0.05, a power equal to 0.80, and Cohen’s d equal to 0.55, the desired sample size was 150 participants in total.

### 2.2. The Experimental Setup

**Figure 1:**
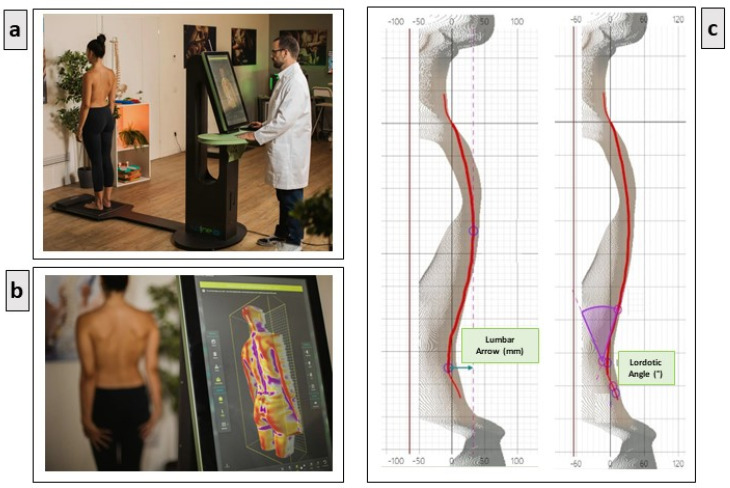
Spine3D non-invasive three-dimensional optoelectronic detection apparatus: (**a**) positioning of the subject during the assessment; (**b**) 3D spine reconstruction; (**c**) sample of software data for lumbar arrow and lordotic angle.

Data were acquired using the Spine3D non-invasive three-dimensional optoelectronic detection system developed by Sensor Medica (Guidonia Montecelio, Italy) [5]; see Figure 1.

This system is equipped with an infrared (IR) time-of-flight (ToF) 3D RGB camera, which is mounted onto a motorized column and controlled via a joystick. The camera has a resolution of 1920 × 1080 pixels at 30 frames per second (fps) for RGB imaging and 512 × 424 pixels at 30 fps for depth resolution, with horizontal and vertical fields of view of 70° and 60°, respectively. The operational measurement range spans from 0.5 to 4.5 m. The ToF camera allows for real-time estimation of the distance between the camera and the participant. Participants were instructed to remove all upper-body clothing and position themselves with their back facing the camera at a distance of 110 cm, ensuring their heels were aligned. They were also asked to lower their pants to expose the intergluteal sulcus. The foot positioning was standardized using custom-designed footprints. Once the participant was in position, with their arms relaxed at their sides, they were directed to focus on a target placed 2 m away to stabilize their posture. The operator then adjusted the camera’s position using a joystick to capture the area from the nape to the glutes. The Spine3D acquisition lasted 7 s. Each acquisition was repeated three times, with a 30 s rest period between repetitions. After each acquisition, the participants stepped away from the camera and then repositioned themselves for the next measurement.

### 2.3. Data Analysis

Upon reconstructing the dorsal surface via time-of-flight (ToF) technology, the software automatically identifies six anatomical landmarks, following the methodology by Ledwon and colleagues [15]. These landmarks include the vertebra prominent (VP), corresponding to the spinous process of C7; the right (SR) and left (SL) acromial apices, located at the midpoint between the superior aspect of the shoulder and the axillary concavity; the right (DR) and left (DL) posterior superior iliac spines (PSISs), with the midpoint between them (DM) also calculated; and the sacral prominence (SP), positioned at the superior aspect of the intergluteal sulcus.

Following landmark identification, several key parameters related to the sagittal profile were computed: (i) the cervical arrow (CA), (ii) the lumbar arrow (LA), (iii) the kyphotic angle (KAn), and (iv) the lordotic angle (LAn). Specifically, the CA is defined as the distance between the most anterior point of the cervical spine and a line perpendicular to the ground, tangent to the apex of the kyphotic curve; LA represents the distance between the most anterior point of the lumbar spine and the same perpendicular line tangent to the kyphotic curve; KAn is the angle formed by the intersection of the tangents at the cervicothoracic and thoracolumbar junctions; and LAn is the angle formed at the intersection of the tangents at the thoracolumbar and lumbosacral junctions [5].

### 2.4. Statistical Analysis

Statistical tests were performed using the Statistical Package for the Social Sciences Software (SPSS), version 12.0 (Chicago, IL, USA), for all of the parametric indexes (CA, LA, KAn, and LAn). Descriptive statistics were assessed for all variables. Before statistical comparisons were made, a Kolmogorov–Smirnov test was performed using SPSS to evaluate the normal distribution of the data. Subsequently, for each group (CTRL, OMT, and GTI), the differences in the indexes or demographic data between S1 and S2 were assessed using a paired *t*-test at S1, as well as at S2, and a repeated-measures ANOVA was adopted to analyze the differences among groups (CTRL vs. OMT vs. GTI), with the treatment as the main within-group factor, followed by Bonferroni’s post hoc test when the ANOVA results reached significance. Statistical significance was considered at *p* < 0.05 (* *p* < 0.05, ** *p* < 0.005, *** *p* < 0.001).

## 3. Results

One-hundred and sixty-five healthy individuals (median age: 28.51 ± 5.77 years; height: 173.64 ± 9.45 cm; weight: 69.43 ± 13.31 kg; forty-six female) were recruited for the experimental protocol and randomized into the CTRL, GTI, and OMT groups. In Table 1, the demographic features of the individuals enrolled into each group are reported.

No one withdrew from the trial, and all of the indexes were collected for the whole cohort of participants at S1 and S2. No statistical differences in the demographic data or in the assessed indexes (CA, LA, KAn, and LAn) were reported among the CTRL, GTI, and OMT groups at S1.

The mean and standard deviation of the values collected at S1 and S2 for the cervical and lumbar arrows in the CTRL, GTI, and OMT groups are reported in Table 2. The statistical significance in the comparison of S1 vs. S2 is also reported in Table 2.

**Figure 2:**
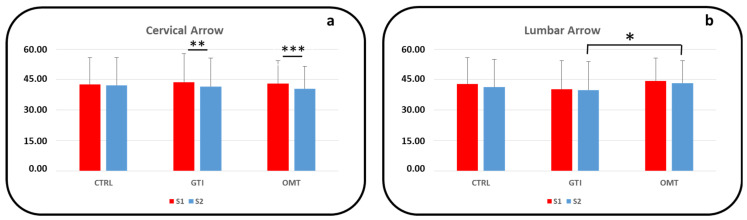
Cervical arrow (**a**) and lumbar arrow (**b**) data at the S1 (red color) and S2 assessments (blue color) are presented in the histograms for the CTRL, GTI, and OMT groups. Statistically significant differences in the S1 vs. S2 comparison are indicated by the lines above columns for the GTI and OMT groups in Figure 1a. Statistically significant differences in the comparison between the GTI and OMT groups at S2 are indicated by the line above the blue columns in Figure 1b (*: *p* < 0.05, **: *p* < 0.005, ***: *p* < 0.001).

For the CTRL group, the CA data did not change between S1 and S2 (see Figure 1a). On the contrary, for the GTI and OMT groups, a significant reduction in the value for CA at S2 compared with that at S1 was enhanced (*p* < 0.005 for the GTI group; *p* < 0.001 for the OMT group). The LA data collected at S2 were only slightly lower than at the initial assessment at S1; indeed, no statistical significance was highlighted (See Figure 2b).

As regards the comparison among the CTRL, GTI, and OMT groups at S1 and at S2, no statistical differences were obtained for the CA data. On the contrary, one statistically significant difference was identified for the LA data between the GTI and OMT groups at the S2 assessment (*p* < 0.05).

The data collected at S1 and S2 for the kyphotic and lordotic angles of the CTRL, GTI, and OMT groups are reported in Table 3. Statistical significance in the comparison S1 vs. S2 is also reported in Table 3.

**Figure 3:**
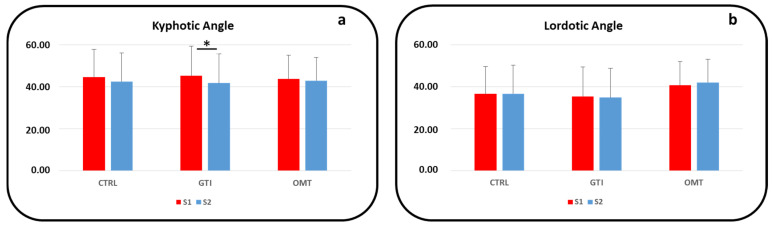
Kyphotic angle (**a**) and lordotic angle (**b**) data at the S1 (red color) and S2 assessments (blue color) are presented in the histograms for the CTRL, GTI, and OMT groups. Statistically significant difference in the S1 vs. S2 comparison for the GTI group is indicated by the line above the GTI columns (*: *p* < 0.05).

The KA data collected at S2 underpin a general pattern of a reduction in values compared to those at the initial assessment for all groups (*p* > 0.05). In detail, this reduction was statistically significant only for the GTI group (*p* < 0.05, see Figure 3a). The data collected at S1 and S2 for the LA did not reveal significant differences for any group due to the almost unchanged values (see Figure 3b). The comparison among the CTRL, GTI, and OMT groups did not reveal any statistical significance for either the KA or LA data.

## 4. Discussion

Our study aimed to investigate the effects of manual therapy interventions on spinal posture in the sagittal plane using the Spine3D non-invasive three-dimensional optoelectronic detection system. This system provided a 3D reconstruction of the participants’ spinal profiles, allowing for an analysis of key postural indexes. As this was a self-controlled three-arm RCT, we ensured that all of the participants were assessed immediately both before (S1) and after (S2) the intervention, facilitating the observation of potential modifications in their postural indexes. In line with Molinaro et al. [5]’s suggestion about the instrument’s reliability, we performed two assessments for each participant, 48 h apart.

The results obtained demonstrated significant changes in specific postural parameters after manual therapy; on the contrary, no changes in the CTRL group were detected. Specifically, the CA index showed a statistically significant reduction in both the GTI and OMT groups, suggesting that this kind of instrumental evaluation is able to objectivate postural changes. Furthermore, both interventions had a measurable effect on cervical posture. These findings are consistent with previous research indicating improvements in postural parameters following manual therapy, particularly in the cervical region [16]. The mechanism according to which these changes occur is still debated and could be due to physical changes in tissue, neural adaptations, or recalibrations in proprioception caused by the interventions.

A key distinction in our study was the comparison between the effects of OMT and GTI. While both interventions produced changes in spinal posture, particularly in the cervical and thoracic regions, the mechanisms and potential clinical implications of these two interventions differ significantly. OMT involves a structured, hands-on approach that targets specific somatic dysfunctions through a range of osteopathic techniques, such as soft tissue manipulation; high-velocity, low-amplitude thrusts; and myofascial release [9]. OMT is designed to directly influence the musculoskeletal, vascular, and neural systems to promote better alignment and function [13]. In our study, the OMT group demonstrated significant reductions in the CA index, indicating an improvement in cervical alignment, and although the LA index showed a trend of reduction, this did not reach statistical significance, however. These results align with the therapeutic goals of OMT, which aims to restore structural balance and optimize body function.

In contrast, GTI involves light, non-invasive contact with the body, with minimal mechanical input [17,18,19]. Despite this, our results show that GTI was able to produce significant reductions in the CA and KAn indexes. The reduction in KAn in the GT group was particularly interesting, as it was statistically significant (*p* < 0.05), while this effect was not observed in the OMT group. This suggests that GTI, despite its more subtle nature, might influence the thoracic curvature by enhancing proprioception or inducing a relaxation response in the superficial tissues. GTI has been thought to promote interoception, which may lead to postural adjustments through neurophysiological pathways rather than direct mechanical effects [20]. In newborns, skin-to-skin contact induces important psychoneuroimmunoendocrine changes, with the production of oxytocin, reductions in acute pain [21], reductions in jaundice disease and increases in good sleep, increases in resilience, and increases in life expectancy [22,23].

The differences between these two interventions, GTI and OMT, suggest that both may work through distinct physiological mechanisms. OMT appears to have a more direct impact on the musculoskeletal structures, targeting specific dysfunctions through manual techniques, while GTI may influence posture by modulating proprioceptive and interoceptive feedback or promoting autonomic changes [24]. Indeed, gentle touch is thought to be mediated by C-tactile (CT) fibers, a type of recently identified unmyelinated, slow-conducting, mechanosensitive nerve located in the skin [25,26]. These fibers are highly responsive to slow, gentle stroking motions, such as light brushing [27]. This hypothesis is supported by the fact that both groups demonstrated improvements in the CA index, yet only the GTI group showed significant changes in the KAn index, highlighting a unique response to this intervention.

The lack of significant findings for the LA index across all groups raises questions about the responsiveness of the lumbar region to manual therapies over short-term intervals. While OMT and GTI both influenced the cervical and thoracic parameters, the lumbar spine may require longer-term intervention or more focused treatment techniques to exhibit statistical and meaningful changes. This observation is consistent with previous research, which often reports more subtle changes in the lumbar curvature compared to those in other regions of the spine following manual therapy [28].

Moreover, our study found no significant changes in LAn in any of the groups, suggesting that the thoracolumbar curvature remains relatively stable in response to short-term interventions. This stability could be due to the biomechanical properties of the lumbar spine, which is generally more resistant to changes due to its role in weight-bearing and overall spinal stability. The non-significant findings in the CTRL group for all of the postural indexes addressed reinforce the notion that the changes observed in the GTI and OMT groups were intervention-specific and not due to natural postural variability. The absence of statistical differences in the demographic characteristics or baseline (S1) postural parameters among the three groups supports the robustness of our study design.

Our findings support the utility of both OMT and GTI as viable manual therapies for influencing spinal posture, particularly in the cervical region. For clinicians, this suggests that both therapies can be effective tools for modulating posture, but they may be appropriate for different therapeutic goals. OMT, with its hands-on, targeted approach, may be more suited to patients with specific musculoskeletal dysfunctions requiring correction, while GTI might be more appropriate for individuals seeking relaxation or subtle postural adjustments without the need for deeper mechanical intervention.

Given that GTI produced significant changes in the kyphotic angle, it could be considered a valuable therapy for patients who are sensitive to more forceful manipulative techniques or who prefer a less invasive approach. Additionally, GTI could serve as a complementary therapy to OMT, especially in cases where manual therapy is contraindicated or when a gentler approach is desirable for promoting neurophysiological balance.

Future studies could explore the potential relationship between modifications in spinal posture and the autonomic nervous system (ANS)’s activity. It is well established that OMT affects the ANS. Several studies [13,29,30,31] have shown that OMT induces a significantly greater parasympathetic response compared to sham or no-touch procedures. Cerritelli and colleagues [32] expanded this evidence, demonstrating that OMT influences heart rate variability parameters alongside facial temperature changes. Similarly, Ioannou and colleagues [33], using high-resolution thermal infrared imaging, reported significant temperature increases in specific facial regions, recognized as proxies for ANS activity following OMT. Considering these findings of OMT’s effects on the ANS, it would be valuable to investigate the relationship between changes in spinal curves and autonomic reactivity further in devoted studies, also because the efficient and balanced use of neural–myofascial–skeletal components may normalize postural responses [34]. Our findings will contribute to a growing body of research that seeks to validate non-invasive posture analysis technologies and enhance our understanding of how manual therapies can modify the spinal alignment in both clinical and non-clinical populations.

### Limitations

As with any study, there are limitations that must be acknowledged. Our cohort consisted of healthy participants without spinal deformities or significant musculoskeletal or neurological disorders. Therefore, the results may not be directly generalizable to clinical populations with conditions such as scoliosis, chronic back pain, or other musculoskeletal disorders. Future research should focus on investigating the effects of OMT and GT in clinical populations to understand their therapeutic potential better. Additionally, while the Spine3D system provides an effective means of analyzing external postural changes, it does not offer insights into the internal anatomical structures of the spine. Consequently, we cannot determine whether the observed postural changes were accompanied by alterations in vertebral alignment or the deeper tissue structures. Future studies incorporating imaging techniques such as MRI could offer a more comprehensive understanding of the effects of manual therapy on both the external and internal anatomy. Lastly, our study assessed only the short-term postural changes following manual therapy, with the follow-up conducted 48 h post-intervention. Long-term follow-up is necessary to ascertain whether the postural improvements observed in this study would be sustained over time and contribute to functional benefits such as pain relief or improved mobility.

## 5. Conclusions

In conclusion, this study suggests that manual therapy interventions may contribute to changes in spinal posture on the sagittal plane, particularly in the cervical and thoracic regions. Both OMT and GTI appear to exert their effects primarily through modulation of the autonomic nervous system. GTI, in particular, emerges as a gentle yet potentially effective approach to influencing thoracic posture. Further research is needed to evaluate the long-term effects of these interventions and their applicability to broader clinical populations, including individuals experiencing pain.

## Figures and Tables

**Table 1:** Demographic features of CTRL, GTI, and OMT groups.

	Age (Mean ± SD)	Gender (M/F)	Weight (Mean ± SD)	Height (Mean ± SD)
CTRL	28.75 ± 5.3	35/20	68.18 ± 12.12	172.98 ± 9.44
GTI	27.55 ± 5.58	32/23	69.45 ± 14.72	173.53 ± 9.8
OMT	27.85 ± 4.17	32/23	69.77 ± 12.06	173.93 ± 8.49

**Table 2:** Cervical and lumbar arrow data for CTRL, GTI, and OMT groups at S1 and S2.

	Cervical Arrow				Lumbar Arrow			
	S1		S2		S1		S2	
	Mean	SD	Mean	SD	Mean	SD	Mean	SD
CTRL	42.65	17.44	42.25	17.80	42.76	14.20	41.31	13.94
GTI	43.62 *	17.52	41.62 *	17.53	40.25	13.62	39.89	14.30
OMT	43.05 *	13.89	40.51 *	14.88	44.42	11.50	43.33	11.99

* = statistical significance difference in the comparison of S1 vs. S2, *p* < 0.05.

**Table 3:** Kyphotic and lordotic angle data for CTRL, GTI, and OMT groups at S1 and S2.

	Kyphotic Angle				Lordotic Angle			
	S1		S2		S1		S2	
	Mean	SD	Mean	SD	Mean	SD	Mean	SD
CTRL	44.60	12.56	42.47	12.25	36.61	13.39	36.62	14.64
GTI	45.23 *	13.32	41.71 *	12.44	35.40	12.08	34.80	11.81
OMT	43.73	11.95	42.89	11.14	40.69	10.58	42.06	12.26

* = statistical significance difference in the comparison of S1 vs. S2, *p* < 0.05.

## Data Availability

The study data can be shared upon reasonable request to the corresponding author.

## Supplementary Materials

The following supporting information can be downloaded at: https://www.mdpi.com/article/10.3390/jcm14020634/s1.
