# Supplementary material for: Three-Dimensional Posture Analysis-Based Modifications After Manual Therapy: A Preliminary Study
Source: J Clin Med. 2025 Jan 19;14(2):634. doi: 10.3390/jcm14020634 (PMC11765545; doi:10.3390/jcm14020634)
Supplement: Supplementary file 1 [file jcm-14-00634-s001.zip › jcm-3392060-supplementary.pdf]

Case Report Form (CRF)

Nome/Cognome: ____________________ **Sesso**: F / M

Data di nascita: ____________ **Età all'arruolamento**: ____________

Peso: ____________ Kg **Altezza**: ____________ cm

Check-list:

- Mini Mental State Examination (MMSE) score: _______

- Presenza di disturbi medici pregressi o attuali:
  - SI, specificare: ____
  - NO

- Presenza di patologie di carattere ortopedico o neurologico pregresse o attuali:
  - SI, specificare quando e cosa: ____
  - NO

- Presenza di dolore alla scala Visual Analogue Scale negli ultimi 6 mesi:
  - SI (indicare il valore: ____/10 e specificare in quale distretto corporeo: ________________)
  - NO

- Nel caso di soggetto di sesso femminile, è in stato di gravidanza? SI NO

- Traumi:
  - SI, specificare quando e cosa: ____
  - NO

- Soggetto fumatore: SI NO

- Utilizzo di Alcol: SI NO

Data Arruolamento: ____________

Data 1° Valutazione: ____________ **Data 2° Valutazione**: ____________
